# The epigenetic factor CHD4 contributes to metastasis by regulating the EZH2/β-catenin axis and acts as a therapeutic target in ovarian cancer

**DOI:** 10.1186/s12967-022-03854-1

**Published:** 2023-01-21

**Authors:** Jieyu Wang, Fangfang Zhong, Jun Li, Huiran Yue, Wenzhi Li, Xin Lu

**Affiliations:** 1 Present Address: Department of Gynecology, Obstetrics and Gynecology Hospital, Fudan University, Shanghai, 200090 China; 2 Shanghai Key Laboratory of Female Reproductive Endocrine-Related Disease, Fudan University, Shanghai, 200090, China; 3 Department of Pathology, Obstetrics and Gynecology Hospital, Fudan University, Shanghai, 200090 China

**Keywords:** Chromodomain-helicase-DNA-binding protein 4, Ovarian cancer, Histone deacetylase inhibitor, Romidepsin, Metastases

## Abstract

**Background:**

The overall survival rate of patients with advanced ovarian cancer (OC) has remained static for several decades. Advanced ovarian cancer is known for its poor prognosis due to extensive metastasis. Epigenetic alterations contribute to tumour progression and therefore are of interest for potential therapeutic strategies.

**Methods:**

Following our previous study, we identified that CHD4, a chromatin remodelling factor, plays a strong role in ovarian cancer cell metastasis. We investigated the clinical significance of CHD4 through TCGA and GEO database analyses and explored the effect of CHD4 expression modulation and romidepsin treatment on the biological behaviour of ovarian cancer through CCK-8 and transwell assays. Bioluminescence imaging of tumours in xenografted mice was applied to determine the therapeutic effect of romidepsin. GSEA and western blotting were used to screen the regulatory mechanism of CHD4.

**Results:**

In ovarian cancer patient specimens, high CHD4 expression was associated with a poor prognosis. Loss of function of CHD4 in ovarian cancer cells induced suppression of migration and invasion. Mechanistically, CHD4 knockdown suppressed the expression of EZH2 and the nuclear accumulation of β-catenin. CHD4 also suppressed the metastasis of ovarian cancer cells and prevented disease progression in a mouse model. To inhibit the functions of CHD4 that are mediated by histone deacetylase, we evaluated the effect of the HDAC1/2 selective inhibitor romidepsin. Our findings indicated that treatment with romidepsin suppressed the progression of metastases in vitro and in vivo.

**Conclusions:**

Collectively, our results uncovered an oncogenic function of CHD4 in ovarian cancer and provide a rationale for clinical trials of romidepsin in ovarian cancer patients.

**Supplementary Information:**

The online version contains supplementary material available at 10.1186/s12967-022-03854-1.

## Background

Ovarian cancer is the most lethal gynaecologic malignancy [1]. Ninety percent of ovarian cancers are epithelial ovarian cancer (EOC), the most common subtype of which is serous carcinoma [2]; this type of cancer is characterized by local aggressiveness and implantation metastasis. Most serous carcinomas are diagnosed at stage III (51%) or IV (29%) [2]. Although remarkable advances have been achieved in surgical techniques and targeted chemotherapy, the 5-year cause-specific survival rate for stage III disease remains at 42% and is further decreased to 26% for stage IV disease [2]. Based on the reality that even optimal treatment has a nominal influence on survival, it is necessary to further explore the molecular mechanisms that are responsible for driving the malignant progression of EOC.

Our lab previously identified 42 invasion-related miRNAs; of these genes, let-7a, let-7f, let-7e, miR-923 and miR-498 target the same gene: chromodomain helicase DNA binding protein 4 (CHD4) [3]. CHD4 is considered a core component of the nucleosome remodelling and histone deacetylation (NuRD) complex [4, 5]. It is believed that CHD4 mainly plays a role in cancer by participating in histone deacetylation [6, 7] and PARP dependent DNA damage repair [8, 9].

CHD4 is a participant in histone deacetylation, and studies have shown that the abnormal expression of CHD4 is related to the occurrence and development of tumours [10]. CHD4 is commonly suggested to act as a tumour suppressor. Nevertheless, emerging evidence points to a role for CHD4 in oncogenic processes, including the epigenetic suppression of tumour suppressor genes [11], cancer metastasis [11], cell-cycle transition [12], cell state determination [13], chemotherapy tolerance [14], and epithelial-to-mesenchymal transition [15].

In recent years, it has been revealed that CHD4 can also protect genome integrity by controlling homologous recombination-mediated repair [16]. CHD4 regulates homologous recombination-mediated DNA repair, and its deficiency sensitizes cells to poly ADP-ribose polymerase (PARP) inhibitor treatment [17]. CHD4 promotes the repair of DNA double-strand breaks (DSBs) and cell survival after DNA damage, mediating rapid PARP-dependent recruitment of the NuRD complex to DNA damage sites [18].

In the present study, we identified that CHD4 is critical for the malignancy of EOC and uncovered that the CHD4/EZH2/β-catenin regulatory circuit enhances the migration and invasion properties of EOC cells. Intriguingly, high CHD4 expression was associated with a poor prognosis. CHD4 also confers antimetastatic effects on ovarian cancer cells and prevents disease progression. Mechanistically, CHD4 knockdown suppressed the expression of EZH2 and the nuclear accumulation of β-catenin. Moreover, we found that romidepsin, a selective HDAC1/2 inhibitor, suppressed the growth of metastases in vitro and in vivo in a dose-dependent manner.

## Material and methods

### Patients and clinical characteristics

In this study, the relationship between CHD4 expression and detailed clinical information was analysed. First, the values of CHD4 expression between 607 tumour tissues and 130 nonneoplastic tissues were extracted from The Cancer Genome Atlas (TCGA) (https://cancergenome.nih.gov/). Next, we evaluated the CHD4 expression level in samples of different pathologic types, and the data were extracted from various independent research datasets in the Gene Expression Omnibus (GEO) (https://www.ncbi.nlm.nih.gov/geo/). Subsequently, the relationship between CHD4 expression level and clinical characteristics was analysed. Finally, combining data from TCGA and GEO, the prognostic significance of the expression profile of CHD4 was evaluated.

### Cell line culture

The human ovarian cancer cell lines SKOV3, Caov3, Hey and OVCAR3 were obtained from the American Type Culture Collection (ATCC, Manassas, VA, USA). SKOV3-ip was kindly gifted by Cui Heng et al. of Pecking University. All cell lines were maintained in RPMI 1640 (Jinuo Co., Ltd, Shanghai, China) medium with 10% foetal bovine serum (Gibco, Life Technologies, Grand Island, NY, USA). Cells were counted with a TC10 Automated Cell Counter (Bio-Rad Laboratories, Inc. Hercules, California, USA). Cells were maintained routinely at 37 °C in a 5% CO_2_ humidified atmosphere.

### Plasmid transfection and lentivirus infection

Knockdown of CHD4 protein expression was achieved by RNA interference with two independent small interfering RNAs (siRNAs, Additional file 1: Table S1). To establish a stable CHD4 knockdown cell line, a lentiviral vector containing a short hairpin RNA (shRNA) was constructed (Additional file 1: Table S1). Furthermore, an expression plasmid containing the full-length EZH2 sequence (Additional file 1: Table S1) was generated, and the corresponding lentiviral vectors were generated by GeneChem (Shanghai, China). Stable clones were obtained by transfecting cells with the corresponding lentiviral vector. The selection medium for transfected cells was supplemented with 1 μg/ml puromycin (Sigma Co., Ltd, USA).

### Cell proliferation assays

SKOV3-ip, SKOV3-ip-siControl, SKOV3-ip-siCHD4-1 and SKOV3-ip-siCHD4-2 cell lines were plated in 96-well plates (Corning, NY, USA) at a density of 1 × 10^3^ cells per well and incubated for 0 h, 24 h, 48 h, 72 h or 96 h at 37 °C. At each indicated time, cell viability was evaluated by the Cell Counting Kit-8 (CCK-8, Dojindo Molecular Technologies Inc., Gaithersburg, MD, USA) assay, followed by incubation for 4 h. The absorbance was measured at 450 nm using a BioTeK Reader. Cell proliferation was tested according to the manufacturer’s protocol. Each experiment was repeated at least three times.

The SKOV3-ip cell line was plated in 96-well plates at a density of 1 × 10^3^ cells per well. After 24 h, the medium was replaced by medium with different concentrations of romidepsin (0 nM, 1 nM, 10 nM, 50 nM and 1 μM, 10 μM, 100 μM, Med Chem Express, New Jersey, USA) for 0 h, 24 h, 48 h, 72 h and 96 h. Romidepsin was dissolved in 0.1% sterile dimethyl sulfoxide (DMSO, Sangon Biotech, Shanghai, China). The control group was treated with 0.1% DMSO solution. At each indicated time point, cell proliferation was evaluated by the CCK-8 assay.

### Cell migration and invasion assays

For the migration assay, the cells were starved overnight with serum-free medium, and then 2 × 10^4^ cells in 100 µl of serum-free 1640 medium with/without 10 nM romidepsin were transferred into the upper compartment of uncoated chambers (Corning, New York, USA). The lower chamber was filled with 500 µl of 1640 medium containing 10% FBS with/without 10 nM romidepsin. For the invasion assay, the insert membranes were precoated with diluted (1:8) Matrigel (Corning, NY, USA). After 12 h of incubation, the cells on the upper surface of the membrane were removed. Cells that passed through the membrane were fixed with 4% paraformaldehyde (Sangon Biotech, Shanghai, China) and stained with 0.1% crystal violet (Beyotime, Shanghai, China). Cells in 5 random fields were counted using an Olympus light microscope at 200× magnification.

### Quantitative reverse transcriptase (qRT)-PCR

Total RNA was extracted using TRIzol (Invitrogen, Carlsbad, CA), and reverse transcription was performed using the Revert Aid First Strand cDNA Synthesis Kit (Fermentas, MA, USA) according to the manufacturer’s instructions. qRT-PCR was performed on the SYBR PrimeScript RT-PCR Kit (TAKARA Bio Inc., Otsu, Shiga, Japan) on an ABI 7900. Data were analysed using SDS 2.3 software, and the threshold cycle (Ct) values were normalized to GAPDH. The mRNA levels were calculated using the equation 2^−ΔΔCt^ based on experiments performed in triplicate. All experiments were performed in triplicate. The primers used for all genes are listed in Additional file 2: Table S2.

### Western blot analysis

To collect total (whole-cell) protein, the cells were lysed with RIPA buffer (Beyotime, Shanghai, China) and 1 mM protease inhibitor phenylmethanesulfonyl fluoride (Beyotime, Shanghai, China), agitated for 15 min at 4 °C, and centrifuged at 12,000 rpm for 25 min. To collect the nuclear protein, the cells were manipulated according to the protocol of the nuclear and cytoplasmic protein extraction kit (Beyotime, Shanghai, China). Protein concentration was measured with a BCA protein assay kit (Beyotime, Shanghai, China). Proteins were electrophoresed on SDS-polyacrylamide gels and transferred onto a polyvinylidene fluoride (PVDF) membrane (Merck KGaA, Darmstadt, Germany). The PVDF membrane was blocked for 1 h at room temperature (RT) in 10% milk powder in PBS with 0.2% Tween. The primary antibodies were diluted and incubated overnight at 4 °C (the primary antibody information is provided in Additional file 3: Table S3). Then, the membranes were washed three times for 10 min in PBS with 0.2% Tween. Afterwards, the membranes were incubated in goat anti-rabbit IgG conjugated with horseradish peroxidase (1:5000 dilution; MT-bio, Shanghai, China) at RT for 1 h. The membranes were washed again six times and incubated with enhanced chemiluminescence (ECL) reagent (Thermo, MA, USA) for visualization. GAPDH and lamin were used as internal controls to normalize the protein expression levels of the whole-cell extract and nuclear extract, respectively.

### Bioluminescent mouse xenograft tumorigenesis studies

All experiments were performed in accordance with the Laboratory Animal Unit (LAU) guidelines and approved by the Committee on the Use of Live Animals in Teaching and Research (CULATR) of The University of Fudan. Stable luciferase-expressing SKOV3-ip cells were resuspended in 100 µl serum-free culture medium and mixed with 100 µl Matrigel (Corning, NY, USA). Six-week-old female BALB/c (nu/nu) mice were intraperitoneally injected with the mixture. After allowing tumour formation for 10 days, the animals were randomized into 3 groups as follows: (i) the control group (treated with an equal volume of 0.1% DMSO); (ii) the low-dose romidepsin group (intraperitoneally injected with 1 mg/kg romidepsin on days 11, 14, 17, 21, 24 and 27); and (iii) the high-dose romidepsin group (intraperitoneally injected with 2 mg/kg romidepsin on days 11, 14, 17, 21, 24 and 27). Baseline imaging data were recorded for all mice on day 10 (start of drug administration) and reimaged posttreatment on days 20 and 30. For imaging acquisition, the mice were anaesthetized with 1.5% pentobarbital (50 µl/10 g, i.p.) and initial imaging of mice was performed with the BRUKER In Vivo Xtreme; after injecting substrate d-luciferin (Science Light. Shanghai, China, 150 mg/kg). Mouse weight was measured every other day to assess the toxicity of the drugs. After 30 days, the nude mice were euthanized by i.p. injection of 200 mg/kg pentobarbital, and the location and number of metastatic foci were determined. Tumour tissue and liver tissue were excised and processed for histology and immunohistochemistry (IHC). The lesions were stored in 4% paraformaldehyde or RNAStay.

### Statistical analysis

Statistical analyses were performed using SPSS 19.0 software. Means and standard deviations (SDs) were calculated for all independent experiments. All values were analyzed by unpaired Student’s t-test. Differences with *p*-values < 0.05 were considered statistically significant.

## Results

### CHD4 expression is associated with ovarian cancer severity and patient survival

**Fig. 1:**
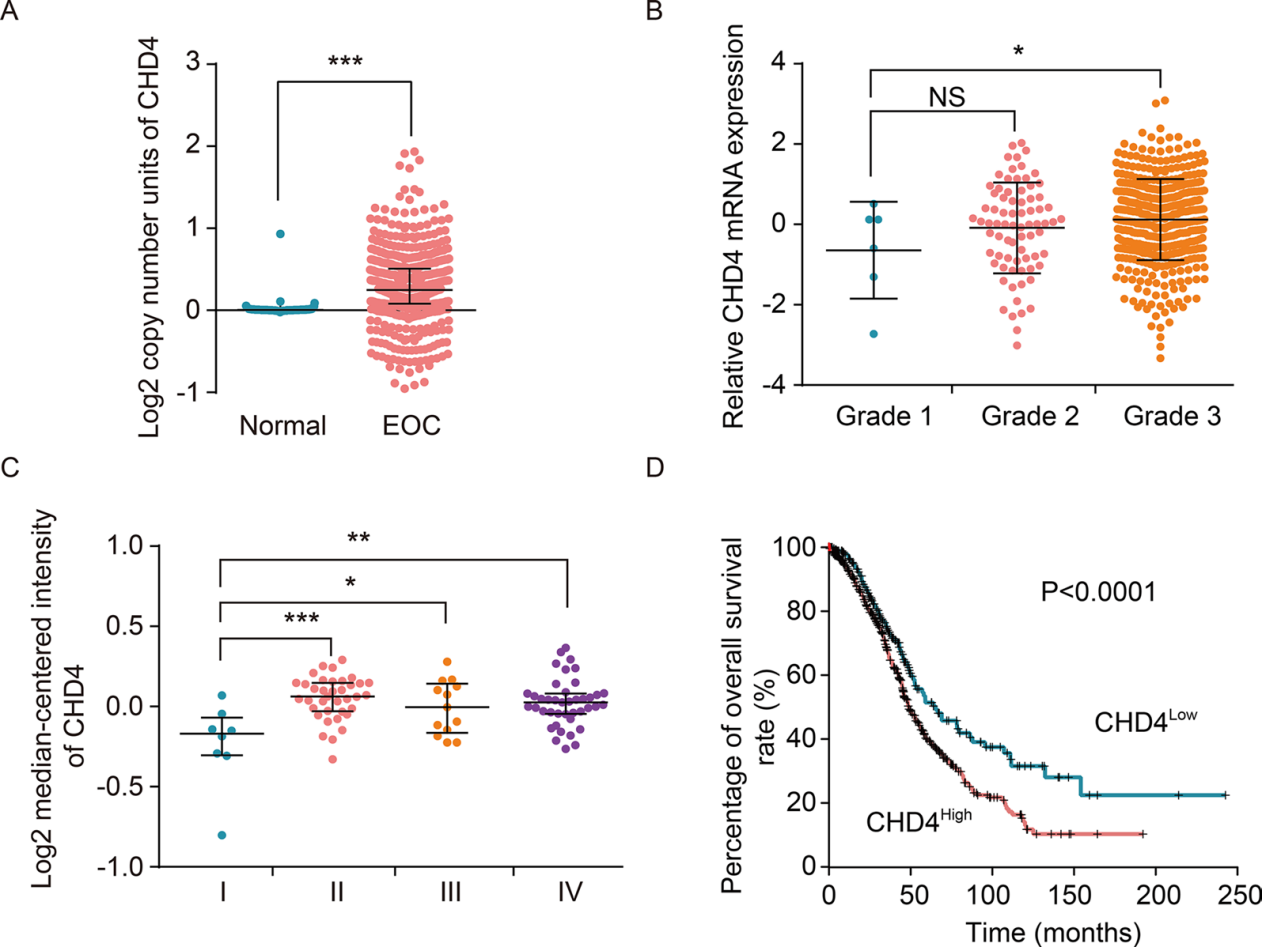
High expression of CHD4 is closely related to poor prognosis of patients with ovarian cancer. **A** The expression level of CHD4 in ovarian cancer tissue was higher than that in normal tissue (P < 0.001); **B** the relationship between the expression level of CHD4 and FIGO stage of serous ovarian cancer patients; **C** the relationship between the expression level of CHD4 and pathological grade of serous ovarian cancer; **D** Kaplan–Meier survival curve analysis showed that the overall survival of patients with high expression of CHD4 was inferior to that of those with low expression of CHD4 (*p* < 0.0001). ***p < 0.0001, **p < 0.01, *p < 0.05

The TCGA cohort of OC patients was used to examine the mRNA expression of CHD4 in ovarian cancer tissue compared to nontumor tissue. CHD4 mRNA expression was 18.9-fold higher in ovarian cancer tissue than in nontumor tissue (*p* < 0.0001; Fig. 1A). Considering that ovarian cancer has various pathologic types, CHD4 mRNA levels in different histologic types were analysed through the GEO database. In some databases, the expression level of CHD4 was not significantly different (Additional file 4: Fig. S1). However, the CHD4 expression level was higher in the serous type than in the clear cell type (*p* = 0.04 in the Meyniel ovarian database) and in the mucous type (*p* = 0.005 in the Lu ovarian database or *p* = 0.0279 in the Meyniel ovarian database or *p* = 0.0001 in the Schwartz ovarian database, Additional file 5: Fig. S2). For this reason, we next focused on serous ovarian cancer. In the serous type, CHD4 mRNA levels correlated with the pathological grade (Fig. 1B) and International Federation of Gynaecology and Obstetrics (FIGO) stage (Fig. 1C) of the disease. Low CHD4 mRNA expression was significantly associated with FIGO stage (stage I vs. stage III; *p* = 0.0018; stage I vs. stage IV; *p* = 0.0010; stage II vs. stage III; *p* = 0.0411; stage II vs. stage IV; *p* = 0.0321) and tumour grade (G; *p* = 0.0331) (Fig. 1B, C). Furthermore, high CHD4 mRNA expression in tumour tissue was significantly associated with poor overall survival (OS) (HR = 1.48; 95% CI 1.18–1.86; *p* < 0.0001; Fig. 1D).

### Reduced CHD4 expression in malignant ovarian cell models decreases cell viability and suppresses aggressive behaviours

**Fig. 2:**
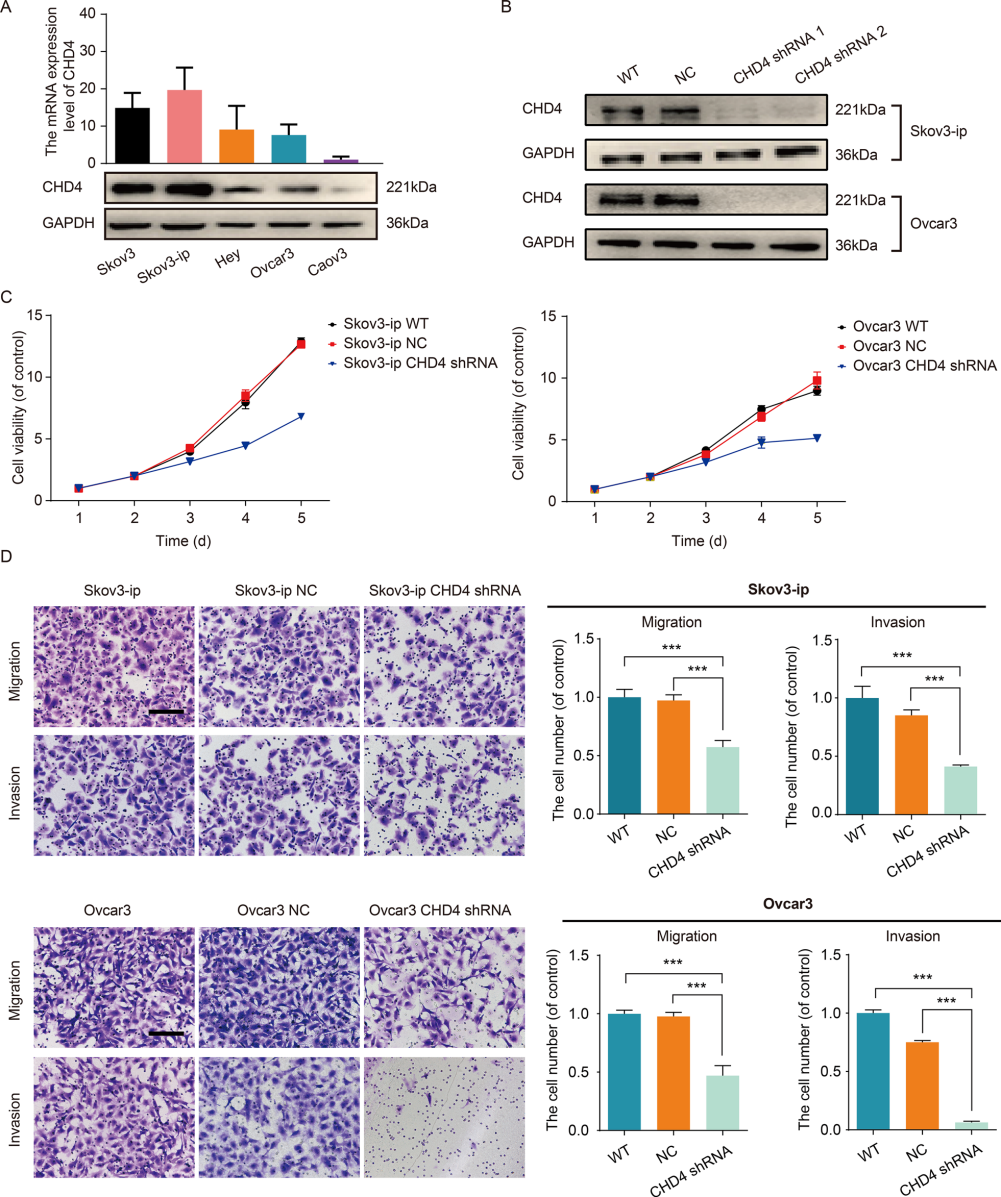
Downregulation of CHD4 expression inhibits the proliferation, invasion and migration of ovarian cancer. **A** The protein expression level of CHD4 in ovarian cancer cell lines; **B** the effect of CHD4 knockdown by shRNAs; **C** downregulation of CHD4 can significantly inhibit the proliferation of ovarian cancer cells; **D** downregulation of CHD4 expression can significantly inhibit the migration and invasion of ovarian cancer cells. ***p < 0.0001, **p < 0.01, *p < 0.05

It is important to consider whether the silencing of CHD4 may influence the biological behaviour of ovarian cancer. To assess the cellular impact of reduced CHD4 expression levels, we first evaluated the baseline mRNA and protein expression levels in some ovarian cancer cell lines (SKOV3, SKOV3-ip, Hey, OVCAR3, and Caov3, Fig. 2A). Considering that CHD4 had a high expression level in SKOV3-ip and OVCAR3, we selected the two cell lines to explore the impact of knocking down CHD4 (Fig. 2B) on cell viability (Fig. 2C), migration, and invasion (Fig. 2D). Notably, CHD4 depletion sharply reduced the viability (*p* < 0.05 at 48 h; *p* < 0.001 at 72 h and 96 h) (Fig. 2C) and metastasis-related behaviour (Fig. 2D) of ovarian cancer cells, suggesting that CHD4 is required to maintain the metastases of ovarian cancer cells. These findings reflect phenotypes observed in CHD4 knockdown cells.

### Overcoming aggressive tumour behaviour by targeting CHD4 through HDAC inhibition

**Fig. 3:**
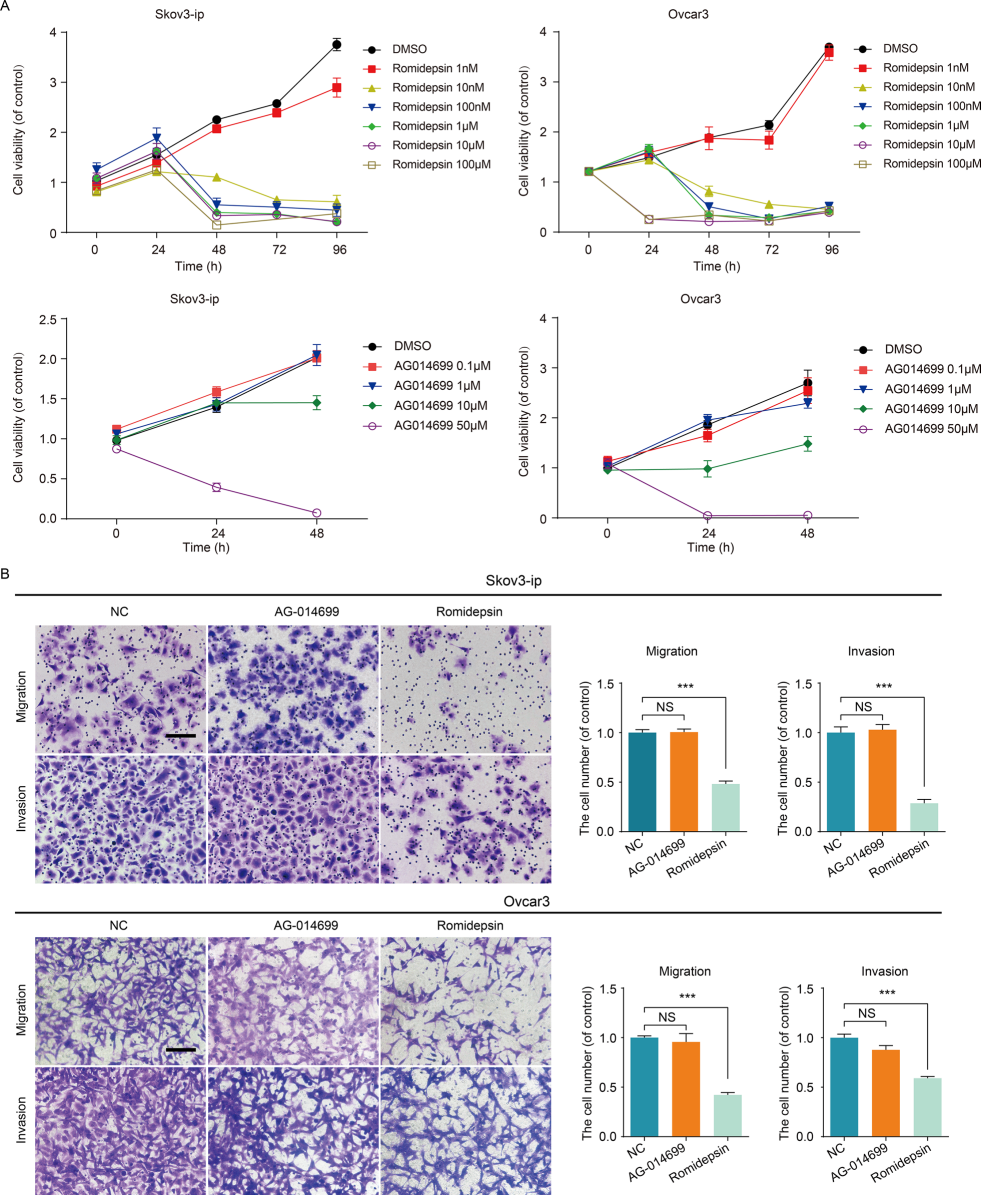
CHD4 regulates the invasion and metastasis of ovarian cancer by participating in histone deacetylation. **A** The effects of romidepsin or AG-014699 on the viability of ovarian cancer cells; **B** the effects of romidepsin or AG-014699 on the migration and invasion of ovarian cancer cells. Ruler: 200 μm. ***p < 0.0001, **p < 0.01, *p < 0.05

The above data demonstrate that the activation of CHD4 may play a major role in the progression of OC, suggesting that CHD4 suppression may be useful for eradicating the metastasis of OC. CHD4 is a core component of the NuRD (nucleosome remodelling and deacetylation) complex. The protein deacetylation role of NuRD is achieved in collaboration with histone deacetylase 1 (HDAC 1) and HDAC2 proteins. On the other hand, there has been a steady accumulation of evidence that CHD4 cooperates with PARP in repairing DSBs [8]. Taken together, these studies imply that CHD4 may support the progression of ovarian cancer by regulating the cellular histone status via HDAC or the DNA damage response via PARP. We evaluated the biological influence of romidepsin (HDAC inhibitor) or AG-014699 (PARP inhibitor) on the ovarian cancer cell lines. Both romidepsin and AG-014699 can decrease the viability of ovarian cancer cells. However, only romidepsin suppressed their migration and invasion (Fig. 3B). Incontrast, no inhibition of migration or invasion was observed with AG-014699 treatment (Fig. 3B). The above findings indicate that the prometastatic ability of CHD4 is independent of PARP but dependent on the normal function of HDAC.

### Romidepsin induced a pronounced dose-dependent antitumour effect in vivo

**Fig. 4:**
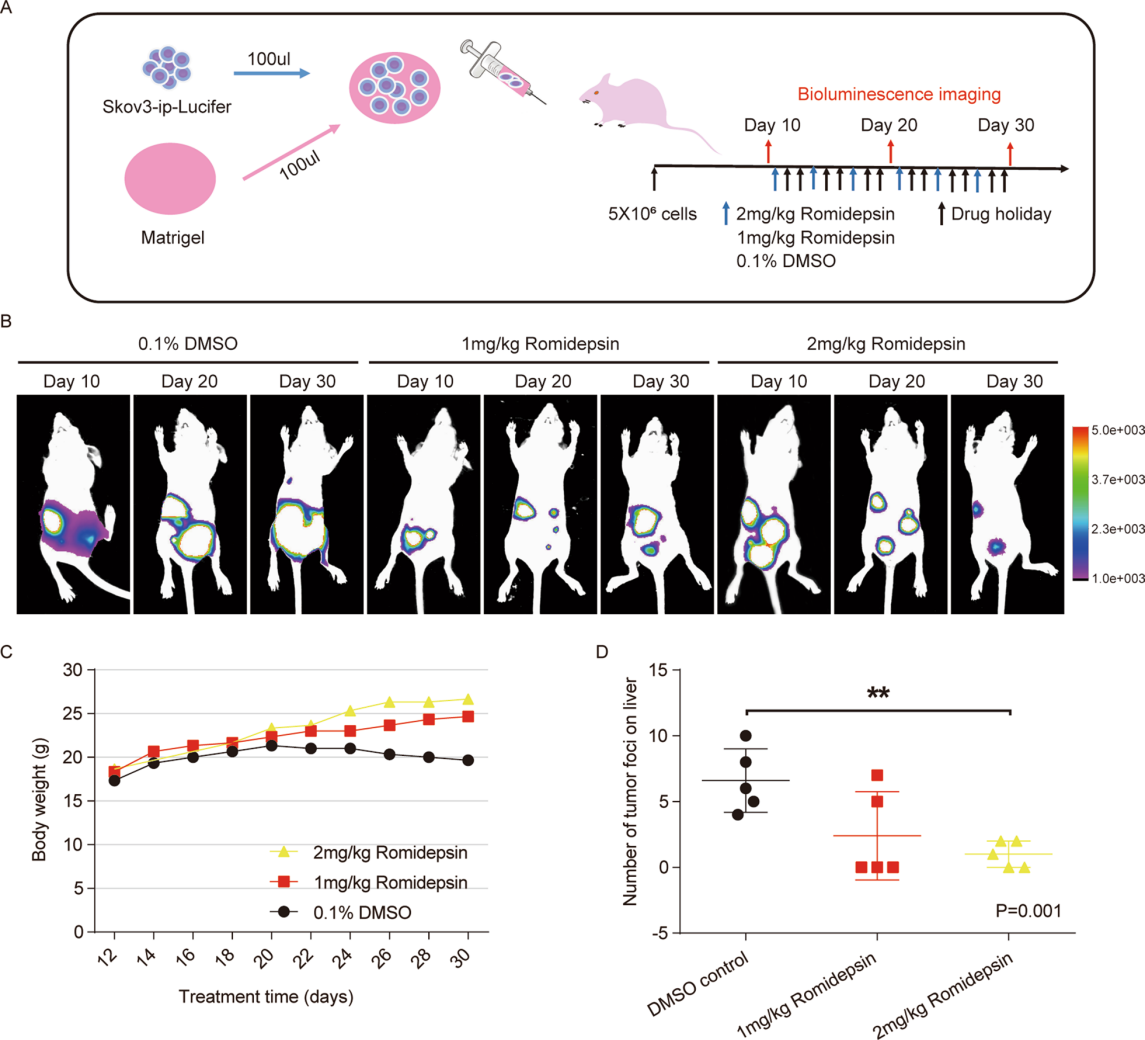
Therapeutic effect of CHD4 inhibitor on ovarian cancer in vivo. **A** The administration mode diagram: ovarian cancer cells and Matrigel were mixed at a ratio of 1:1 and injected intraperitoneally into mice. The growth of the tumour was observed by an in vivo imaging instrument on the 10th day after injection, and then different drugs were administered on the 1st, 4th, 7th, 11th, 14th and 17th days. The control group was given 0.1% DMSO, the low-dose group was given 1 mg/kg romidepsin, and the high-dose group was given 2 mg/kg romidepsin. Imaging was performed every 10 days to observe the growth of the tumour. **B** Bioluminescence imaging of tumour bearing nude mice after treatment with 0.1% DMSO, low-dose romidepsin, and high-dose romidepsin. **C** The weights of nude mice in the romidepsin treatment group was higher than that in the DMSO control group. **D** The number of liver metastases in nude mice in the high-dose romidepsin treatment group was significantly less than that in the DMSO control group. **p < 0.01

HDACs are significant biological targets for the development of efficient anticancer therapeutics. However, pan-inhibition of HDACs is less efficient than isoform-selective inhibition. HDAC1 and HDAC2 are associated with various cellular events responsible for cancer growth and development. Based on the above findings, as an inhibitor of HDAC1 and HDAC2, romidepsin suppresses cell migration and invasion in vitro. We evaluated the therapeutic effect of romidepsin on OC cells in vivo using bioluminescence imaging of a mouse model. First, we implanted OC cells through intraperitoneal injected and allowed them to grow for 10 days to establish the tumours. Mice were then randomized to groups and treated with the control, low-dose romidepsin (1 mg/kg) or high-dose romidepsin (2 mg/kg) by i.p. injection on days 11, 14, 17, 21,24 and 27 after tumour cell inoculation (Fig. 4A). Indeed, high-dose romidepsin treatment significantly inhibited the metastasis of OC tumours (Fig. 4B). In contrast, the inhibitory role of low-dose romidepsin was barely satisfactory (Fig. 4B). We concluded that high-dose treatment with the selective HDAC inhibitor romidepsin significantly suppressed the growth of OC tumours. Mouse body weight was significantly higher in the romidepsin groups than in the control group 22 days after tumour inoculation (Fig. 4C). Furthermore, there were significantly fewer liver metastasis lesions in the high-dose group than in the DMSO control group (Fig. 4D). Together, these data support a model in which romidepsin inhibits the metastasis of OC tumours.

### EZH2/β-catenin may be a potential pathway by which CHD4 regulates the invasion and metastasis of ovarian cancer

**Fig. 5:**
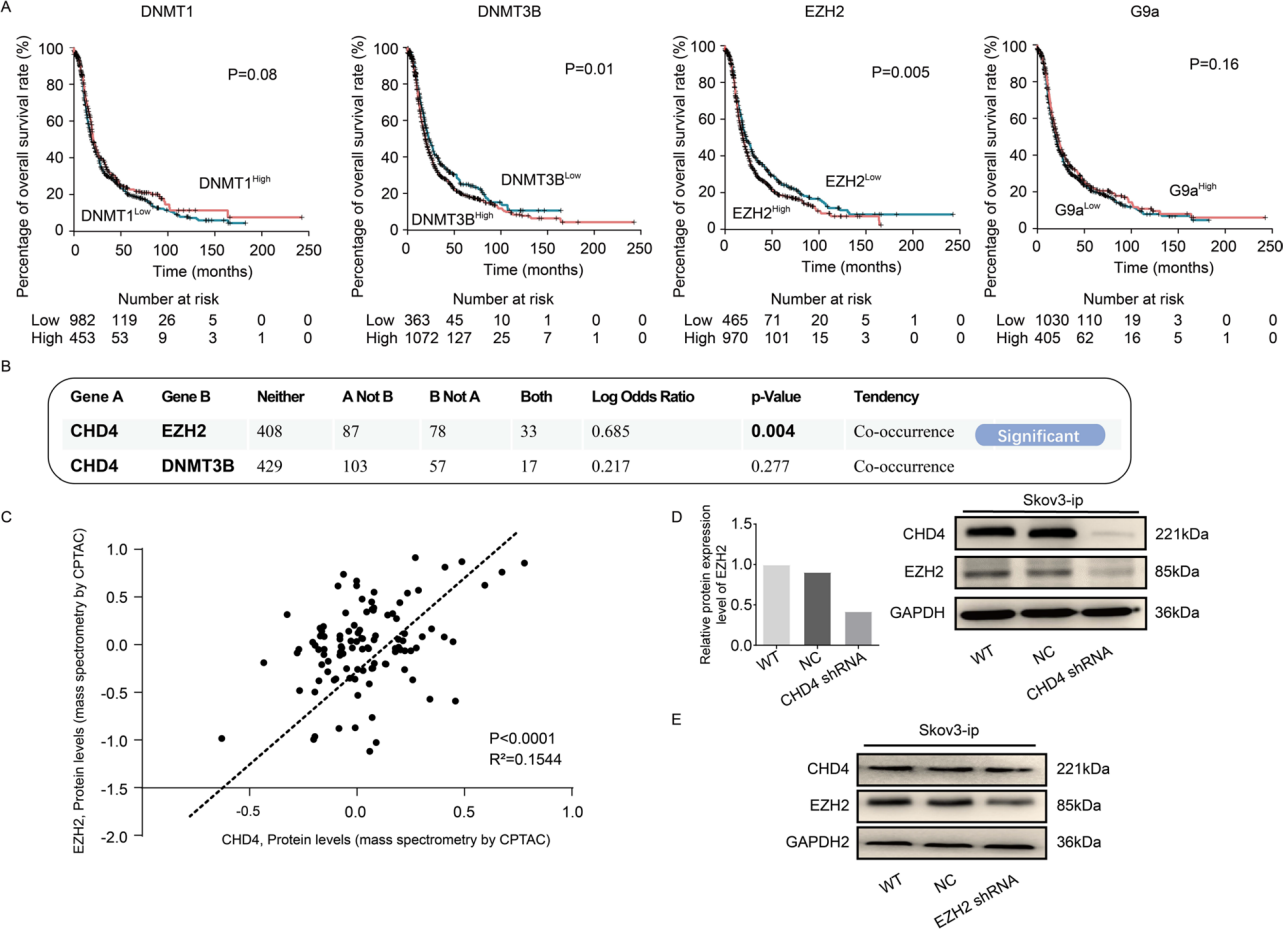
The downstream molecule of CHD4 is EZH2. **A** The relationship of the expression levels of DNMT1, DNMT3b, EZH2 and G9a with the prognosis of patients with ovarian cancer. **B** Analysis of the correlation of CHD4 with EZH2 and DNMT3b at the mRNA expression level. **C** There was a positive correlation between CHD4 and EZH2 at theprotein level (*p* < 0.0001). **D** Downregulation of CHD4 in ovarian cancer cells resulted in decreased EZH2 expression. **E** Western blot analysis showed that inhibition of EZH2 expression had no effect on the expression level of CHD4

A previous study reported that CHD4 could recruit repressive chromatin proteins (DNMT1, DNMT3B, G9a, and EZH2) [11, 19]. We then investigated the prognostic importance of these methylation-associated genes (Fig. 5A). We next analysed the relationship between CHD4 and methylation enzymes (DNMT1, DNMT3B, EZH2, and G9a) in ovarian cancer. We did not observe a significant difference in survival in patients grouped by G9a expression (Fig. 5A). However, DNMT1 expression was inversely related to survival (Fig. 5A). Surprisingly, high expression of DNMT3B and EZH2 was related to poor survival (Fig. 5A). Considering that CHD4 expression was positively correlated with aggressive tumour behaviour, we further focused on DNMT3B and EZH2. Furthermore, we investigated the relationships among EZH2, DNMT3B and CHD4. According to RNA-seq analyses, CHD4 was positively correlated with EZH2 in ovarian cancer patients (*p* = 0.004, Fig. 5B). However, there was no correlation between CHD4 and DNMT3B (*p* = 0.277, Fig. 5B). Although both CHD4 and EZH2 play roles in epigenetics, it has not been determined whether the two modifiers cooperate during cancer progression. Therefore, to investigate the interaction between CHD4 and EZH2, we further analysed the correlation between EZH2 and CHD4 at the protein level. As expected, CHD4 showed a significant positive correlation with EZH2 (Fig. 5C). Based on these findings, we focused our subsequent research on the relationship between CHD4 and EZH2. The clinical significance of EZH2 prompted us to experimentally assess whether CHD4 and EZH2 are correlated in ovarian cancer. For this purpose, we examined the EZH2 protein level in CHD4 knockdown ovarian cancer cells. Notably, the EZH2 expression level decreased significantly when CHD4 was knocked down (Fig. 5D). However, the downregulation of EZH2 did not affect the expression of CHD4 (Fig. 5E). These studies suggest that EZH2 may be a downstream molecule of CHD4 in ovarian cancer cell lines.

### The upregulation of EZH2 reverses the inhibitory effect of CHD4 deletion

**Fig. 6:**
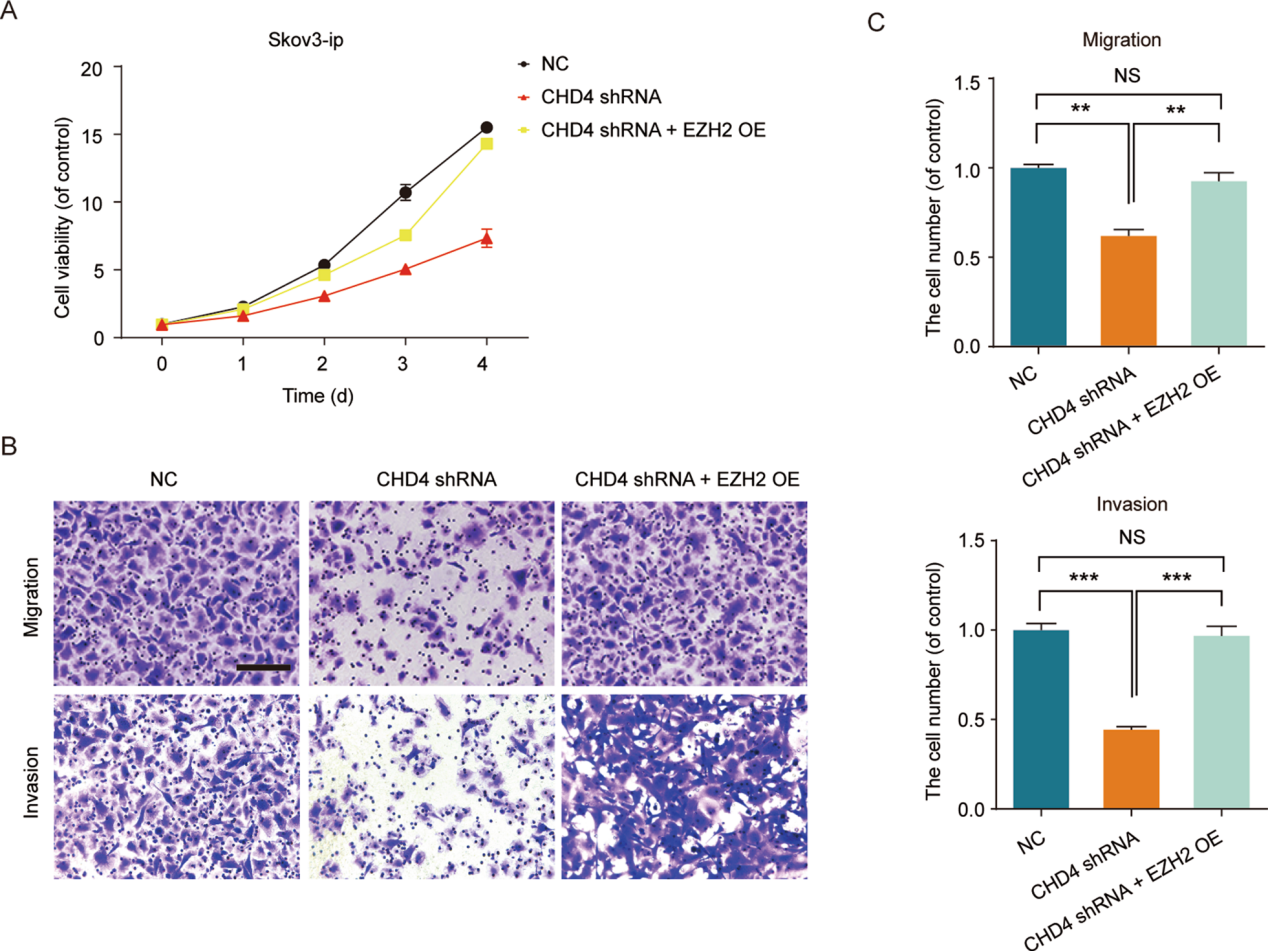
Overexpression of EZH2 reverses the inhibitory effect of CHD4 knockdown on the metastasis of ovarian cancer. **A** Cell viability of Skov3-ip-shControl, Skov3-ip-shCHD4, and Skov3-ip-shCHD4 + EZH2 OE cells. **B**, **C** Migration and invasion ability of Skov3-ip-shControl, Skov3-ip-shCHD4 and Skov3-ip-shCHD4 + EZH2 OE cells. Ruler: 200 μm. ***p < 0.0001, **p < 0.01

The bioinformatics results of CHD4 and EZH2 prompted us to investigate the rescue effect of EZH2. To explore whether EZH2 is necessary for the inhibitory effect of CHD4 on ovarian cancer, we overexpressed EZH2 in a CHD4 knockdown OC cell line. Intriguingly, overexpression of EZH2 significantly attenuated the inhibitory effects of CHD4 knockdown on viability (Fig. 6A) migration and invasion (Fig. 6B). Taken together, these studies suggest that downregulation of CHD4 mediates the inhibition of invasion and metastasis in OC and that EZH2 could reverse the inhibitory effect of CHD4 depletion.

### CHD4 cooperates with EZH2 to induce the nuclear accumulation of β-catenin to promote ovarian cancer progression

**Fig. 7:**
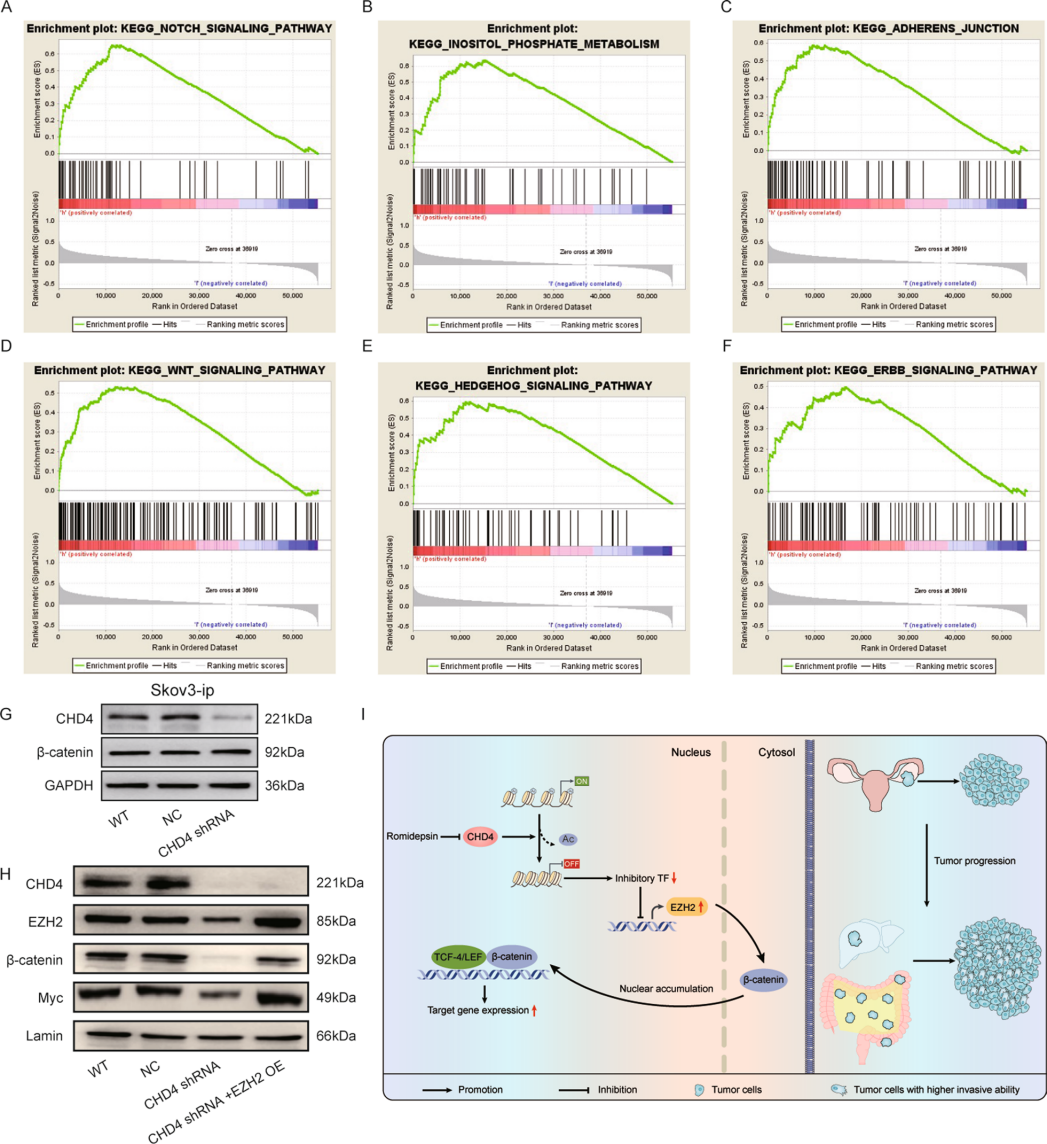
CHD4 is involved EZH2-mediated regulation of the Wnt/β-catenin pathway. **A**–**F** GSEA showed that CHD4 expression was associated with some signalling pathways. **G** The accumulation of total β-catenin did not change when CHD4 was downregulated. **H** Overexpression of EZH2 can reverse the inhibition of β-catenin accumulation in the nucleus caused by the downregulation of CHD4. **I** Diagram of the mechanism by which CHD4 activates the EZH2/β-catenin pathway to promote ovarian cancer invasion and metastasis

According to the above results, we preliminarily confirmed that EZH2 plays an important role in the cancer-promoting effects of CHD4. However, the signalling pathway by which CHD4 promotes cancer is still unclear. To solve this problem, we obtained ovarian cancer data from the TCGA for gene set enrichment analysis (GSEA) analysis. The GSEA results showed that high expression of CHD4 was positively correlated with the Notch, Wnt and Hedgehog signalling pathways (Table 1, Fig. 7A–F). A previous study confirmed that EZH2 could regulate Wnt/β-catenin signalling pathways [20, 21]. The results of the GSEA and those of previous studies drew our attention to the Wnt/β-catenin signalling pathway. Nuclear β-catenin accumulation is a sign of Wnt/β-catenin pathway activation. The level of nuclear β-catenin was examined when CHD4 was knocked down. Inconsistent with the whole-cell level results (Fig. 7G), the accumulation of nuclear β-catenin was inhibited when CHD4 was downregulated, and the overexpression of EZH2 reversed this phenomenon (Fig. 7H). These results suggest that EZH2 itself [20] may regulate the nuclear accumulation of β-catenin (Fig. 7I). Further studies are needed to investigate the degree of methylation of β-catenin.

**Table 1:** The pathways positively correlated with CHD4 in TCGA were analysed by gene set enrichment analysis

Signaling name	Size	NES	NOM p-val	FDR q-val	FWER p-val	Rank at max
NOTCH_SIGNALING_PATHWAY	47	2.101641	0	0.010654	0.006	11,320
INOSITOL_PHOSPHATE_METABOLISM	54	2.050652	0	0.016514	0.016	15,207
BASAL_CELL_CARCINOMA	55	2.034353	0	0.012997	0.018	10,713
ADHERENS_JUNCTION	73	2.017389	0.002004	0.012376	0.028	9599
PHOSPHATIDYLINOSITOL_SIGNALING_SYSTEM	76	1.979099	0.001992	0.015025	0.039	15,207
WNT_SIGNALING_PATHWAY	150	1.978311	0.002037	0.012521	0.039	12,401
TASTE_TRANSDUCTION	51	1.940397	0	0.017843	0.054	16,175
HEDGEHOG_SIGNALING_PATHWAY	56	1.872574	0.00404	0.039105	0.13	12,001
LYSINE_DEGRADATION	44	1.855039	0.003937	0.042479	0.154	13,292
MELANOGENESIS	101	1.838217	0.006	0.047405	0.184	16,175
ERBB_SIGNALING_PATHWAY	87	1.809339	0.005882	0.057518	0.222	16,540
COLORECTAL_CANCER	62	1.807526	0.008032	0.053987	0.227	10,941
ENDOMETRIAL_CANCER	52	1.803657	0.008032	0.051899	0.231	10,941
THYROID_CANCER	29	1.756727	0.008163	0.076846	0.313	14,471
PROSTATE_CANCER	89	1.753645	0.010225	0.0733	0.316	11,645
GNRH_SIGNALING_PATHWAY	101	1.749633	0.006061	0.070386	0.32	16,894
AXON_GUIDANCE	129	1.739887	0.014028	0.072038	0.339	16,047
SPLICEOSOME	127	1.727772	0.022	0.075285	0.356	6232
UBIQUITIN_MEDIATED_PROTEOLYSIS	134	1.722847	0.01222	0.074673	0.369	12,985

## Discussion

Invasion and metastasis are the main obstacles in the treatment of OC. Abnormal epigenetic regulation is closely related to the invasion and metastasis of ovarian cancer cells [22]. An imbalance in histone acetylation can lead to oncogene activation and tumour suppressor gene silencing [23], which is closely related to the occurrence and development of a variety of cancers [24]. These studies indicate that intervening in histone acetylation should inhibit the invasion and metastasis of OC cells in patients. These findings build upon our previous evidence that CHD4, a central gene in the NuRD complex, is an essential gene for the invasion and metastasis of ovarian cancer [3]. In the current study, we presented the mechanisms by which CHD4, in the context of the NuRD complex, induces a robust metastasis-promoting effect by modulating Wnt/β-catenin signalling and highlighted the therapeutic significance of CHD4 as a novel oncogene in ovarian cancer.

CHD4 is an integral component of the NuRD complex that has both ATP-dependent chromatin remodelling abilities and histone deacetylase activity [25]. In recent years, studies have shown that the abnormal expression of CHD4 is related to the development of tumours, but it was previously unclear whether it acts as a tumour suppressor gene [26] or an oncogene [27]. In endometrial cancer, CHD4 is involved in the progression from endometrial atypical hyperplasia (AH) to uterine endometrioid carcinoma (EC) [28, 29], and mutations in CHD4 can promote endometrial tumorigenesis [30]. Regarding prognosis, CHD4 is associated with poor clinical outcomes [31]. In colorectal cancer (CRC) cells, CHD4 is one of the most significantly upregulated genes among all subunits of the NuRD complex [32]. CHD4 overexpression is associated with poor prognosis in CRC [32], papillary thyroid carcinoma (PTC) [33] and breast cancer [34]. In ovarian cancer, our findings verified that the expression level of CHD4 is associated with the pathological grade and FIGO stage. In the population of patients with optimal cytoreductive surgery, CHD4 is associated with inferior prognosis, which lays a clinical foundation for our further research.

CHD4 has previously been demonstrated to be required for the metastasis and invasion of cancer cells [35]. In thyroid cancer, deleterious variants of CHD4 were found to be restricted to metastatic lesions [36]. In triple-negative breast cancer (TNBC) and PTC cell lines, CHD4 was significantly associated with the metastatic state [33, 37] and mediated epithelial-mesenchymal transition (EMT) [33, 38]. Knockdown of CHD4 in human hepatocellular carcinoma (HCC) cells inhibited cell migration and invasion [27]. Downregulation of CHD4 could reduce the proliferative and migratory ability of non-small cell lung cancer (NSCLC) cells and could decrease tumorigenicity in nude mice [39]. In the present study, CHD4 knockdown inhibited cell invasion and decreased metastasis compared to that in the control groups. All these results demonstrate that CHD4 acts as an oncogene in ovarian cancer. Accordingly, we showed that CHD4 could inhibit the metastasis of ovarian cancer using a mouse model.

CHD4 is a catalytic subunit of the NuRD complex, but recent studies have also demonstrated that CHD4 is rapidly recruited to sites of DNA damage in a PARP-dependent manner [9, 40]. Certainly, these findings indicate that CHD4 may have an oncogenic role in ovarian cancer. However, it remains to be seen whether this role is realized via the PARP pathway or the histone deacetylation pathway. AG-014699 treatment further suppresses the recruitment of CHD4 during the DNA damage response, and romidepsin, an HDAC inhibitor (HDACi) [41], is a cyclic peptide that mainly inhibits HDAC1 and HDAC2 enzyme activity, which synergizes with CHD4 deacetylation activity during histone deacetylation. In vitro, we found that romidepsin suppresses the prometastatic effect of CHD4 in ovarian cancer, which indicates that the prometastatic effect of CHD4 may be realized via histone deacetylation.

Romidepsin was approved by the Food and Drug Administration (FDA) for the treatment of patients with relapsed or refractory cutaneous T-cell lymphoma (CTCL) and peripheral T-cell lymphoma (PTCL) [42]. The number of studies on romidepsin in solid tumours is increasing. The therapeutic role of romidepsin has been studied in bladder cancer [43], osteosarcoma [44], esophageal squamous cell carcinoma (ESCC) [45], HCC [46, 47], urothelial carcinoma [48] and lung cancer [49]. However, its possible effectiveness in the treatment of OC remains unknown. The concentration and duration of romidepsin treatment are also under discussion. In the treatment of T-cell lymphoma, romidepsin was injected intraperitoneally at 1.2 mg/kg and 2 mg/kg at 1, 8 and 14 days [50]. Hui et al. used 375 µg/kg or 750 µg/kg doses of romidepsin (i.p., days 1 and 4 of each week) over 4 weeks for the treatment of nasopharyngeal carcinoma [51, 52]. Zheng et al. used 2 mg/kg (on days 14, 16 and 18) as the dose for treating lung adenocarcinoma [49]. In malignant T-cell lymphoma, Makena was intravenously injected with 1.25 mg/kg romidepsin on days 1, 4, 8, 11 and 15 (with a cycle length of 21 days and a total of 2 cycles) [53]. Sawa et al. used 0.5 µg/g as the dose (i.p. injection on days 8 and 12) for the treatment of glioblastoma [54]. Intravenous injection of 1 mg/kg romidepsin was previously tested in lymphoma cells [55]. Our findings demonstrate that the therapeutic effect of 2 mg/kg romidepsin in the ovarian cancer mouse model was more obvious than that of 1 mg/kg romidepsin, and we will explore this further as we develop our studies.

Another area for further study is the underlying mechanism by which CHD4 functions in OCs. Interestingly, in the context of its epigenetic role, studies have confirmed that CHD4 can interact with the methylation enzymes DNMT1, DNMT3B, EZH2 and G9a in cancer progression [11, 19]. It is therefore conceivable that methylation enzymes might play a role in CHD4-dependent tumorigenesis inhibition. However, G9a, a histone methyltransferase, shows no apparent value as a survival-related factor in ovarian cancer. These data in combination with our data suggesting that CHD4 has a promoting effect on ovarian cancer indicate that DNMT1 is unlikely to be a contributing factor in this context due to its anticarcinogenic effect. Based on the survival analysis, we narrowed the scope of study to DNMT3B and EZH2. Correlation analysis showed that CHD4 and EZH2 were positively correlated at the mRNA and protein levels, while DNMT3B was not correlated with these factors. However, ablation of CHD4 at the time of EZH2 overexpression has been shown to reverse the inhibitory effect of CHD4. Here, we identified EZH2 as a cofactor for CHD4 and established the physiological significance of its interaction with CHD4 in the context of ovarian cancer progression. Overall, we observed that EZH2 is essential for CHD4-mediated repression of ovarian cancer tumorigenesis.

In addition, we further used GSEA to analyse the potential pathway involved in CHD4-mediated inhibition of ovarian cancer. CHD4 was found to be closely related to the Wnt/β-catenin, Notch and Hedgehog signalling pathways. Previous studies have demonstrated that EZH2 can regulate the Wnt/β-catenin pathway directly or indirectly. In HCC, EZH2 directly mediates methylation of the β-catenin protein at the K49 site, inhibits its degradation via ubiquitination, and then promotes its nuclear accumulation [20]. On the other hand, EZH2 can also promote the methylation of GSK3β, ICAT and AXIN2 and then indirectly regulate the activity of β-catenin [21]. Therefore, we speculate that CHD4 cooperates with EZH2 repression via β-catenin. Our present study showed that the accumulation of nuclear β-catenin decreased when CHD4 was downregulated. Furthermore, overexpression of EZH2 partially reversed the inhibition of β-catenin nuclear accumulation induced by CHD4 knockdown. These results suggest that EZH2 may interact with β-catenin to promote the nuclear accumulation of β-catenin, which leads to the enhancement of ovarian cancer cell invasion and migration.

## Conclusions

In summary, our results highlight a novel and essential role for CHD4 in the maintenance of ovarian cancer in vitro and in vivo. We also validated the essential role of CHD4 in patient samples. Herein, our data indicate that CHD4 cooperates with EZH2 in ovarian cancer to exert its key roles and that these roles may be mediated in part by Wnt/β-catenin pathway activation. As subunits of the NuRD complex, CHD4 and HDAC1/2 exert synergistic effects on histone deacetylation. The HDAC1/2i romidepsin attenuates the prometastatic effect of CHD4 in ovarian cancer, which indicates that the prometastatic effect of CHD4 may be realized via histone deacetylation. To date, no studies with romidepsin have been conducted for ovarian cancer, and we propose that such preliminary exploration should be a high priority, especially given the dose-dependent effect of this treatment in metastatic ovarian cancer. However, the regulation mechanism and the markers which can predict the sensitivity to romidepsin need to be further explored through in vivo model to guide the clinical application of romidepsin.

## Supplementary Information

**Additional file 1: Table S1.** Information about CHD4-siRNA, CHD4-shRNA and EZH2-overexpression sequence.

**Additional file 2: Table S2.** The primer sequences of CHD4 and GAPDH.

**Additional file 3: Table S3.** The antibody information.

**Additional file 4: Figure S1.** CHD4 expression levels in the Denkert, Etemadmognadam, Ramakrishna, and Schaner epithelial ovarian cancer related datasets. **A** The expression level of CHD4 in Denkert epithelial ovarian cancer related dataset; **B** The expression level of CHD4 in Etemadmognadam epithelial ovarian cancer related dataset; **C** The expression level of CHD4 in Ramakrishna epithelial ovarian cancer related dataset; **D** The expression level of CHD4 in Schaner epithelial ovarian cancer related dataset.

**Additional file 5: Figure S2.** CHD4 expression levels in Hendrix, Lu, Meyniel, and Schwartz epithelial ovarian cancer related dataset. **A** The expression level of CHD4 in Lu epithelial ovarian cancer related dataset. The expression level of CHD4 in ovarian endometrioid adenocarcinoma epithelial ovarian cancer was higher than that in mucinous adenocarcinoma (*p* = 0.0247), and the expression level of CHD4 in ovarian serous adenocarcinoma was significantly higher than that in mucinous adenocarcinoma (*p* = 0.005); **B** The expression level of CHD4 in Meyniel epithelial ovarian cancer-related dataset. The expression level of CHD4 in ovarian serous adenocarcinoma was significantly higher than that in mucinous adenocarcinoma (*p* = 0.0279) and ovarian clear cell adenocarcinoma (*p* = 0.04); **C** The expression level of CHD4 in Schwartz epithelial ovarian cancer related dataset. The expression level of CHD4 in mucinous adenocarcinoma was significantly lower than that in ovarian clear cell adenocarcinoma (*p* = 0.0114), ovarian endometrioid adenocarcinoma (*p* = 0.0027) and ovarian serous adenocarcinoma (*p* = 0.0001). ***p < 0.0001, **p < 0.01, *p < 0.05.

## Data Availability

The datasets generated and analysed during the current study are available in the TCGA data portal repository, https://www.cancer.gov/about-nci/organization/ccg/research/structural-genomics/tcga.

## Abbreviations

OC: Ovarian cancer
EOC: Epithelial Ovarian Cancers
CHD4: Chromodomain-helicase-DNA-binding protein 4
EZH2: Enhancer Of Zeste 2 Polycomb Repressive Complex 2
NuRD: Nucleosome Remodeling and the Histone Deacetylation
PARP: Poly ADP-ribose polymerase
DSBs: DNA double-strand breaks
TCGA: The Cancer Genome Atlas
GEO: Gene Expression Omnibus
CCK-8: Cell counting kit
qRTPCR: Real-time quantitative reverse transcription PCR
PVDF: Polyvinylidene fluoride
RT: Room temperature
ECL: Enhanced chemiluminescence
LAU: Laboratory Animal Unit
CULATR: Committee on the Use of Live Animals in Teaching and Research
SDs: Standard deviations
HDAC: Histone deacetylase
DNMT1: DNA Methyltransferase 1
DNMT3B: DNA Methyltransferase 3B
GSEA: Gene set enrichment analysis
EC: Endometrioid carcinoma
CRCs: Colorectal cancer
PTC: Papillary thyroid carcinoma
TNBC: Triple-negative breast cancer
EMT: Epithelial–mesenchymal transition
NSCLC: Non-small cell lung cancer
HDACi: HDAC inhibitor
FDA: Food and Drug Administration
CTCL: Cutaneous T-cell lymphoma
PTCL: Peripheral T-cell lymphoma
ESCC: Esophageal squamous cell carcinoma
HCC: Human hepatocellular carcinoma
