# Supplementary material for: The epigenetic factor CHD4 contributes to metastasis by regulating the EZH2/β-catenin axis and acts as a therapeutic target in ovarian cancer
Source: J Transl Med. 2023 Jan 21;21:38. doi: 10.1186/s12967-022-03854-1 (PMC9862813; doi:10.1186/s12967-022-03854-1)
Supplement: Supplementary file 1 — Additional file 1: Table S1. Information about CHD4-siRNA, CHD4-shRNA and EZH2-overexpression sequence. [file 12967_2022_3854_MOESM1_ESM.docx]

Table S1. Information about CHD4-siRNA, CHD4-shRNA and EZH2-overexpression sequence.

| Name | Sequence |
| --- | --- |
| siRNA-1 (si1) | 5'-CCCAGAAGAGGAUUUGUCA- 3' |
| siRNA-2 (si2) | 5'-GGUUUAAGCUCUUAGAACA-3' |
| shRNA-F | GATCCGGCGGGAGTTCAGTACCAATAACTCGAGTTATTGGTACTGAACTCCCGCCTT TTTT |
| shRNA-R | AATTAAAAAAGGCGGGAGTTCAGTACCAATAACTCGAGTTATTGGTACTGAACTCCC GCCg |
| EZH2-overexpression sequence | GGCTTTTTTGTTAGACGAAGCTTGGGCTGCAGGTCGACTCTAGAGGATCCCGCCACCATGGGCCAGACTGGGAAGAAAT  CTGAGAAGGGACCAGTTTGTTGGCGGAAGCGTGTAAAATCAGAGTACATGCGACTGAGACAGCTCAAGAGGTTCAGAC  GAGCTGATGAAGTAAAGAGTATGTTTAGTTCCAATCGTCAGAAAATTTTGGAAAGAACGGAAATCTTAAACCAAGAATG  GAAACAGCGAAGGATACAGCCTGTGCACATCCTGACTTCTGTGAGCTCATTGCGCGGGACTAGGGAGTGTTCGGTGAC  CAGTGACTTGGATTTTCCAACACAAGTCATCCCATTAAAGACTCTGAATGCAGTTGCTTCAGTACCCATAATGTATTCTT  GGTCTCCCCTACAGCAGAATTTTATGGTGGAAGATGAAACTGTTTTACATAACATTCCTTATATGGGAGATGAAGTTTT  AGATCAGGATGGTACTTTCATTGAAGAACTAATAAAAAATTATGATGGGAAAGTACACGGGGATAGAGAATGTGGGT  TTATAAATGATGAAATTTTTGTGGAGTTGGTGAATGCCCTTGGTCAATATAATGATGATGACGATGATGATGATGGAG  ACGATCCTGAAGAAAGAGAAGAAAAGCAGAAAGATCTGGAGGATCACCGAGATGATAAAGAAAGCCGCCCACCTC  GGAAATTTCCTTCTGATAAAATTTTTGAAGCCATTTCCTCAATGTTTCCAGATAAGGGCACAGCAGAAGAACTAAAGG  AAAAATATAAAGAACTCACCGAACAGCAGCTCCCAGGCGCACTTCCTCCTGAATGTACCCCCAACATAGATGGACCA  AATGCTAAATCTGTTCAGAGAGAGCAAAGCTTACACTCCTTTCATACGCTTTTCTGTAGGCGATGTTTTAAATATGACTG  CTTCCTACATCGTAAGTGCAATTATTCTTTTCATGCAACACCCAACACTTATAAGCGGAAGAACACAGAAACAGCTCTA  GACAACAAACCTTGTGGACCACAGTGTTACCAGCATTTGGAGGGAGCAAAGGAGTTTGCTGCTGCTCTCACCGCTGAG  CGGATAAAGACCCCACCAAAACGTCCAGGAGGCCGCAGAAGAGGACGGCTTCCCAATAACAGTAGCAGGCCCAGCA  CCCCCACCATTAATGTGCTGGAATCAAAGGATACAGACAGTGATAGGGAAGCAGGGACTGAAACGGGGGGAGAGAA  CAATGATAAAGAAGAAGAAGAGAAGAAAGATGAAACTTCGAGCTCCTCTGAAGCAAATTCTCGGTGTCAAACACCAAT  AAAGATGAAGCCAAATATTGAACCTCCTGAGAATGTGGAGTGGAGTGGTGCTGAAGCCTCAATGTTTAGAGTCCTCATT  GGCACTTACTATGACAATTTCTGTGCCATTGCTAGGTTAATTGGGACCAAAACATGTAGACAGGTGTATGAGTTTAGAGT  CAAAGAATCTAGCATCATAGCTCCAGCTCCCGCTGAGGATGTGGATACTCCTCCAAGGAAAAAGAAGAGGAAACACC  GGTTGTGGGCTGCACACTGCAGAAAGATACAGCTGAAAAAGGACGGCTCCTCTAACCATGTTTACAACTATCAACCCT  GTGATCATCCACGGCAGCCTTGTGACAGTTCGTGCCCTTGTGTGATAGCACAAAATTTTTGTGAAAAGTTTTGTCAATGT  AGTTCAGAGTGTCAAAACCGCTTTCCGGGATGCCGCTGCAAAGCACAGTGCAACACCAAGCAGTGCCCGTGCTACCTG  GCTGTCCGAGAGTGTGACCCTGACCTCTGTCTTACTTGTGGAGCCGCTGACCATTGGGACAGTAAAAATGTGTCCTGCAA  GAACTGCAGTATTCAGCGGGGCTCCAAAAAGCATCTATTGCTGGCACCATCTGACGTGGCAGGCTGGGGGATTTTTATCA  AAGATCCTGTGCAGAAAAATGAATTCATCTCAGAATACTGTGGAGAGATTATTTCTCAAGATGAAGCTGACAGAAGAGGG  AAAGTGTATGATAAATACATGTGCAGCTTTCTGTTCAACTTGAACAATGATTTTGTGGTGGATGCAACCCGCAAGGGTAAC  AAAATTCGTTTTGCAAATCATTCGGTAAATCCAAACTGCTATGCAAAAGTTATGATGGTTAACGGTGATCACAGGATAGGT  ATTTTTGCCAAGAGAGCCATCCAGACTGGCGAAGAGCTGTTTTTTGATTACAGATACAGCCAGGCTGATGCCCTGAAGTAT  GTCGGCATCGAAAGAGAAATGGAAATCCCTGGTATGGACTACAAGGATGACGATGACAAGGATTACAAAGACGACGATG  ATAAGGACTATAAGGATGATGACGACAAATGAGCTAGCACATAACTTACGGTAAATGGCCCGCCTGGCTGACCGCCCAAC  GACCCCCGCCCATTGACGTCAATAGTAACGCCAATAGGGACTTTCCATTGACGTCAATGGGTGGAGTATTTACGGTAAACT  GCCCACTTGGCAGTACATCAAGTGTATCATATGCCAAGTACGCCCCCTATTGACGTCAATGACGGTAAATGGCCCGCCTGG  CATTGTGCCCAGTACATGACCTTATGGGACTTTCCTACTTGGCAGTACATCTACGTATTAGTCATCGCTATTACCATGGTCGAGGTGAGCCCC  Note: The red font is the insertion sequence, the black font is the vector sequence, and the underline marks the enzyme digestion site |
