# Supplementary material for: The epigenetic factor CHD4 contributes to metastasis by regulating the EZH2/β-catenin axis and acts as a therapeutic target in ovarian cancer
Source: J Transl Med. 2023 Jan 21;21:38. doi: 10.1186/s12967-022-03854-1 (PMC9862813; doi:10.1186/s12967-022-03854-1)
Supplement: Supplementary file 2 — Additional file 2: Table S2. The primer sequences of CHD4 and GAPDH. [file 12967_2022_3854_MOESM2_ESM.docx]

Table S2. The primer sequences of CHD4 and GAPDH.

| **Gene** | **Primer** | **Sequence** |
| --- | --- | --- |
| CHD4-1 | Forward | CACCGAATCCTCAACCA |
|  | Reverse | GGCTGTCGCTCATACTT |
|  |  |  |
| CHD4-2 | Forward | CTATGACTCCCTGCTGGTCTTC |
|  | Reverse | AATCCTCTTCACCACCTCCATA |
|  |  |  |
| GAPDH | Forward | CGGAGTCAACGGATTTGGTCGTAT |
|  | Reverse | AGCCTTCTCCATGGTGGTGAAGAC |
