# Supplementary material for: The epigenetic factor CHD4 contributes to metastasis by regulating the EZH2/β-catenin axis and acts as a therapeutic target in ovarian cancer
Source: J Transl Med. 2023 Jan 21;21:38. doi: 10.1186/s12967-022-03854-1 (PMC9862813; doi:10.1186/s12967-022-03854-1)
Supplement: Supplementary file 3 — Additional file 3: Table S3. The antibody information. [file 12967_2022_3854_MOESM3_ESM.docx]

Table S3. The antibody information.

| Antibody name | Corporate name | Dilution ratio |
| --- | --- | --- |
| CHD4 | Abcam Co., Ltd, Shanghai, China | 1:200 |
| GAPDH | Abcam Co., Ltd, Shanghai, China | 1:5000 |
| EZH2 | Cell signaling technology, Boston, USA | 1:1000 |
| Lamin | Abcam Co., Ltd, Shanghai, China | 1:2000 |
| β-catenin | Abcam Co., Ltd, Shanghai, China | 1:5000 |
| Myc | Abcam Co., Ltd, Shanghai, China | 1:10000 |
